# Clinical, biochemical, neuroradiological and molecular characterization of Egyptian patients with glutaric acidemia type 1

**DOI:** 10.1007/s11011-019-00422-3

**Published:** 2019-05-06

**Authors:** Hatem Zayed, Hamed El Khayat, Hoda Tomoum, Ola Khalifa, Ehab Siddiq, Shaimaa A. Mohammad, Radwa Gamal, Zumin Shi, Ahmed Mosailhy, Osama K. Zaki

**Affiliations:** 1 Department of Biomedical Sciences, College of Health Sciences, Qatar University, Doha, Qatar; 2 Medical Genetics Unit, Pediatric Department, Faculty of Medicine, Ain Shams Pediatrics Hospital, Ain-Shams University, Cairo, 11665 Egypt

**Keywords:** Glutaric acidemia type 1, Glutaryl-CoA dehydrogenase, Macrocephaly, Genotype-phenotype correlations, Organic acidemia, Egypt

## Abstract

Glutaric acidemia type 1 (GA1) is an inherited metabolic autosomal recessive disorder that is caused by a deficiency in glutaryl-CoA dehydrogenase (GCDH). Untreated patients suffer primarily from severe striatal damage. More than 250 variants in the *GCDH* gene have been reported with a variable frequency among different ethnic groups. In this study, we aimed to characterize 89 Egyptian patients with GA1 and identify the variants in the 41 patients who were available for genotyping. All of our patients demonstrated clinical, neuroradiological, and biochemical characteristics that are consistent with a diagnosis of GA1. All patients presented with variable degrees of developmental delay ranging from mild to severe. Most of the 89 patients presented with acute onset type (71.9%), followed by insidious (19%) and asymptomatic (9%). A delay in diagnosis was inversely associated with macrocephaly. The prevalence rate ratio (PR) for macrocephaly that was associated with each 6-month delay was 0.95 (95%CI 0.91–0.99). However, high body weight was associated with a higher likelihood of having macrocephaly (PR 1.16, 95%CI 1.06–1.26 per 1 SD increment of Z score weight). However, body weight was inversely associated with the morbidity score. Consanguinity level was 64% among our patient’s cohort and was positively associated with the C5DC level (β (95%CI) 1.06 (0.12–1.99)). Forty-one patients were available for genotyping and were sequenced for the *GCDH* gene. We identified a total of 25 variants, of which the following six novel variants were identified: three missense variants, c.320G > T (p.Gly107Val), c.481C > T (p.Arg161Trp) and c.572 T > G (p.Met191Arg); two deletions, c.78delG (p.Ala27Argfs34) and c.1035delG (p.Gly346Alafs*11); and one indel, c.272_331del (p.Val91_Lys111delinsGlu). All of the novel variants were absent in the 300 normal chromosomes. The most common variant, c.*165A > G, was detected in 42 alleles, and the most commonly detected missense variant, c.1204C > T (p.Arg402Trp), was identified in 29 mutated alleles in 15/41 (34.2%) of patients. Our findings suggest that GA1 is not uncommon organic acidemia disease in Egypt; therefore, there is a need for supporting neonatal screening programs in Egypt.

**Electronic supplementary material:**

The online version of this article (10.1007/s11011-019-00422-3) contains supplementary material, which is available to authorized users.

## Introduction

Glutaric aciduria type 1 (GA1, MIM#231670) is an autosomal recessive inborn error of metabolism. The combined worldwide frequency of GA1 that was calculated from newborn screening of 2.5 million children using MS/MS is 1:100,000 infants (Lindner et al. 2004). This disease is caused by a deficiency of the enzyme glutaryl-CoA dehydrogenase GCDH (Kolker et al. 2006). The *GCDH* gene (NM_000159.3; 19p13.2) consists of 11 exons that span approximately 7-kb of genomic DNA (Biery et al. 1996). GCDH is made as a precursor protein of 438 amino acids. After it is imported into the mitochondria, its 44 N-terminal amino acid residues are cleaved off (Biery et al. 1996; Zschocke et al. 2000). GCDH encodes a flavin adenine dinucleotide-dependent mitochondrial matrix protein that is responsible for the degradative metabolism of L-lysine, L-hydroxylysine, and L-tryptophan. A deficiency of the GCDH enzyme causes the excessive buildup of glutaric acid (GA), 3-hydroxyglutaric acid (3-OH-GA), glutaconic acid, and glutarylcarnitine (C5DC) in various body organs but primarily in the brain (Lindner et al. 2004; Kolker et al. 2011).

There is marked variation in the clinical expression and severity GA1, even within families (Haworth et al. 1991). Most patients present with acute encephalopathy at infancy, and this is often triggered by infection or other minor illness. The outcome is often poor, with a previously well child suffering from spastic cerebral palsy, choreoathetosis, dystonia and, occasionally, mental retardation, although the standard intellectual function is preserved (Brismar and Ozand 1995). Macrocephaly is a very frequent finding in these patients and is present at birth or develops in the first weeks of life (Kolker et al. 2006). In neonates and infants, unspecific neurologic symptoms such as muscular hypotonia and delayed motor development occur in approximately half of all individuals with GA1, whereas other patients are asymptomatic. The clinical presentations of GA1 include macrocephaly; acute encephalopathic crises, which are accompanied by degeneration of the striatum and bilateral marked enlargement of the Sylvian fissure and developmental regression; and frontotemporal atrophy. Untreated patients develop dystonia during infancy, which is reported to be frequent in patients who have had a previous encephalopathic crisis (Kolker et al. 2006), and this is associated with elevated morbidity and mortality.Biochemically, GA1 is characterized by an accumulation of glutaric acid (GA), 3- hydroxyglutaric acid (3-OH-GA), glutaconic acid (less frequently), and glutarylcarnitine (C5DC). These organic acids can be detected in plasma, urine, CSF, and tissues by GC/MS or MS/MS (Baric et al. 1999). GA1 presents with two phenotypes; patients are defined as having a low or high excretor phenotype, according to their levels of urinary glutaric acid excretion (Baric et al. 1999). Interestingly, a comparable risk for developing striatal damage was observed among both low and high excreting patients (Christensen et al. 2004; Kolker et al. 2006), indicating that the genotype-phenotype correlation among patients with GA1 cannot solely rely on the level of GA secretion. Therefore, molecular genetic analyses may serve as a reliable confirmatory molecular diagnostic tests by identifying variants in the *GCDH* gene (Greenberg et al. 2002). To date, over 250 variants have been reported in the Human Gene Mutation Database (HGMD; www.hgmd.cf.ac.uk). The kind and frequency of disease-causing variants vary among different ethnic groups (Biery et al. 1996; Zschocke et al. 2000).

Patients with GA1 receive mainly metabolic treatment, and in case of acute inter-current illness, an intensified emergency treatment is required. The metabolic treatment includes a low lysine diet and carnitine supplementation. Most patients remain asymptomatic if treatment is started in the newborn period (Strauss et al. 2003; Heringer et al. 2010), which proven to be effective in preventing the disease (Kolker et al. 2007a). The three dietary treatment, including low lysine diet, carnitine, and emergency treatment, demonstrated the best outcome in treatment of GA1 patients (Kolker et al. 2007a), compared to the basic metabolic diet treatment (low lysine diet and carnitine), which demonstrated intermediate outcome (Heringer et al. 2010).

In this study, we investigated the clinical, biochemical, and neuroradiological parameters for 89 patients with GA1, and 41 patients were molecularly characterized. This study is expected to serve as a platform for improving genetic counseling and patients’ care in Egypt.

## Methods

### Subjects

This work was performed after approval was obtained from the ethics committee of Ain Shams University and after obtaining a written consent from the patients/patients’ guardians. We studied 89 patients from 2010 to 2018. However, only 41 patients were available for genotyping. The patients were diagnosed with GA1 based on their clinical presentation, neuroimaging and biochemical measurements, and molecular genetic analyses (41 patients) at the Genetics Unit, Ain Shams University Hospital (GUASH). For an objective evaluation of the severity of the clinical condition, the morbidity score was calculated as described previously (Kolker et al. 2006). One point was added for each of the following parameters: loss of mobility, feeding problems, respiratory problems, and seizures. The scores ranged from 0 to 4, according to the number of morbidity parameters that were present in our patient cohort. All our patients received a lysine-restricted diet that was customized according to the recommended guidelines (Boy et al. 2017). We used free lysine and low tryptophan diet formula (XLys-LowTry, Nutricia Inc., USA) in combination with limited amounts of natural proteins with regular clinical follow up of nutritional status and growth. Patients also received L-carnitine supplementation (100 mg/kg/day) to maintain normal plasma concentration of free carnitine. Treatment of other associated neurological manifestations such as dystonia, convulsions as well as metabolic crisis followed the recommended guidelines (Boy et al. 2017).

## Genetic analysis

The ethics committee at Ain-Shaams University, Cairo, Egypt, approved the genetic analysis of 41 patients with GA1. In addition, the parents/guardians signed a written consent. Total RNA was isolated from the whole blood of our patients’ cohort. The QuantiTect Reverse Transcription Kit (Qiagen, Belgium), was used for cDNA synthesis as per the manufacture instructions. The cDNA was PCR amplified using GoTaq Green polymerase Mix 2× (Promega, Fitchburg, WI). The primers used for GCDH cDNA amplification were as follow: forward: 5′-TTGCTCCGCTCGCTCTGAGA G-3′, and reverse: 5′-GCCCATAGGCCACAGACGAA-3′, for the second amplicon: forward: 5′-GATGGGGGAGTTGG GTGT-3′, and reverse: 5′-TGATGATCATGCCTGTGG-3′, for the third amplicon: forward 5′-GTGTGAAGATGGCT GCATTC-3′, and reverse: 5′-GGGCGTGAATGTCA TGTGTA-3′, and for the fourth amplicon were forward: 5′-GGAATGGGATTTCTGACGAG-3′, and reverse: 5′-GGGGTCAGATGTGCAGGTCTTT-3′. The GCDH gene was bi-directionally sequenced using the sequencing service of GATC Biotech, Germany.

## Biochemical analysis

### Tandem mass spectrometry (MS/MS)

A finger prick blood sample was taken from each patient on Guthrie card (Whatman 903 filter paper (GE Healthcare, New Jersey, USA). Acylcarnitines and amino acids were analyzed by triple-quadruple tandem mass spectrometer (ACQUITY UPLC H-Class. Waters® corporation, Massachusetts, USA), with a positive electrospray ionization probe, utilizing Mass Chrom® Amino acids and Acylcarnitines from Dried Blood kit (Chromsystems Instruments & Chemicals GmbH, München, Germany). The data of Multiple Reaction Monitoring (MRM) scan were analyzed using Neolynx® application (Waters® Corporation, Massachusetts, USA).

### Gas chromatography/mass spectrometry (GC/MS)

Patients’ urine samples were collected and frozen until derivatization by silylation of organic compounds. The GC instrument, Agilent 7890 system, interfaced with MS and a gas chromatography capillary column HP 5 MS (Agilent, USA) was used to perform the urine profiling. The results were calculated in μmol/mmol creatinine using a calibration curve of the organic acid of interest (Moseilhy et al. 2016).

## Neuroradiological analysis

An MRI of the brain was performed on the patient at the age of 12 months using the 1.5 T magnet (Achieva, Philips Health Care, Eindhoven, Netherlands). Multiple pulse sequences were acquired. Images of the axial T1-WI, T2-WI and FLAIR along with sagittal T1-WI were obtained. Multiple gray and white matter structures were scored as previously described (Mohammad et al. 2015). Each gray matter structure (caudate, putamen, globus pallidus, substantia nigra and dentate nucleus) was scored as 1 if it displayed only an abnormal T2 signal and scored 2 if there was atrophy. White matter abnormalities were described as confluent or multifocal patches. Their distribution either in the subcortical, central or periventricular location were demonstrated in various cerebral lobes. The white matter score was calculated as a sum of the one-point score for each abnormal white matter zone.

## Statistical analysis

MRI scores (gray and white matter scores) were correlated to morbidity score using a Spearman’s rank-order rho test. Moreover, the MRI scores of patients with and without neonatal screening were compared.

The association between sample characteristics and macrocephaly was assessed using a Poisson regression with robust variance. Logistic regression was used to assess the association between sociodemographic factors, morbidity score and C5DC level. The results were visually presented using a user-written command, *coefplot,* in Stata 15.

## Computational analysis

The stability and pathogenicity of the missense variants in the GCDH protein were predicted using three stability (I-Stable, MuPro, and I-Mutant) and two pathogenicity (SIFT and Polyphen2) predictors. The conservation of amino acids was predicted using the Consurf server. All in silico prediction analysis was done as published earlier (Mosaeilhy et al. 2017).

## Results

### Clinical, biochemical, and neuroradiological findings

Eighty-nine Egyptian patients (60 males and 29 females) were diagnosed with GA1 in the GUASH in the previous eight years (2010–2018). The age of these patients ranges from 28 to 252 months (mean 84.03 ± 39.24) (Table 1), and 52% were referred to the hospital from the Upper Egypt area while 52% were referred from the Delta region. The consanguinity level was 64% (Table 1). The patient diagnoses were clinically, biochemically, and neuroradiologically confirmed in the 89 patients (Supplementary Tables 2–6). The MRI brain scans demonstrated that the globus pallidus structure of the basal ganglia was the most affected area of the brain in 88.6% of patients. This was followed by damage to the putamen and caudate in 83% and 63% of patients, respectively. The central tegmental tract was abnormal in 71% of the patients. Hemispheric white matter was assessed in 17 patients. Confluent and multifocal patches of abnormal signals are observed in the affected individuals. The central white matter zone of both the frontal and parietal lobes were the most frequently involved regions in 14 patients (82%), followed by the periventricular zone of the frontal and parietal lobes in 76% and 65% of patients, respectively. The gray matter score was significantly correlated with the gray matter score (r: 0.6, *p* value: <0.001). The gray matter score was significantly lower in the patients who received neonatal screening, compared to the patients who did not (p value <0.03).

**Table 1:** Summary of all the clinical characteristics of the 89 patients in the supplementary tables. Data are presented as the means (SD) or medians (IQR) for continuous measures, and n (%) for categorical measures

	Male	Female	Total	*p* value
	*N* = 60	*N* = 29	*N* = 89	
Age (month)	82.5 (59.0–99.0)	84.5 (57.0–104.0)	82.5 (59.0–100.0)	0.85
Weight z score	−0.7 (2.1)	−1.1 (1.7)	−0.9 (2.0)	0.44
Residence				0.65
Delta	28 (47%)	15 (52%)	43 (48%)	
Upper Egypt	32 (53%)	14 (48%)	46 (52%)	
Family history	21 (35%)	8 (28%)	29 (33%)	0.48
Consanguinity	39 (65%)	18 (62%)	57 (64%)	0.79
Macrocephaly	44 (73%)	17 (59%)	61 (69%)	0.16
Diagnosis delay (month)	6 (1–11)	7 (2–12)	6 (1–12)	0.64
Type of onset				0.34
Acute	41 (68%)	23 (79%)	64 (72%)	
Insidious	14 (23%)	3 (10%)	17 (19%)	
Screening	5 (8%)	3 (10%)	8 (9%)	
Onset-associated intercurrent infection	41 (68%)	23 (79%)	64 (72%)	0.28
Sibling death	11 (18%)	5 (17%)	16 (18%)	0.90
Tone				0.62
Hyper	36 (60%)	18 (62%)	54 (61%)	
Hypo	19 (32%)	7 (24%)	26 (29%)	
Normal	5 (8%)	4 (14%)	9 (10%)	
Gross motor delay				0.87
Mild	9 (15%)	4 (14%)	13 (15%)	
Moderate	15 (25%)	6 (21%)	21 (24%)	
Severe	36 (60%)	19 (66%)	55 (62%)	
Fine motor delay				0.38
Mild	6 (10%)	6 (21%)	12 (13%)	
Moderate	31 (52%)	13 (45%)	44 (49%)	
Severe	23 (38%)	10 (34%)	33 (37%)	
Speech delay				0.59
Mild	10 (17%)	7 (24%)	17 (19%)	
Moderate	28 (47%)	14 (48%)	42 (47%)	
Severe	22 (37%)	8 (28%)	30 (34%)	
Cognitive delay				0.37
Mild	52 (87%)	23 (79%)	75 (84%)	
Moderate	8 (13%)	6 (21%)	14 (16%)	
Convulsion	23 (38%)	13 (45%)	36 (40%)	0.56
Intracranial hemorrhage	4 (7%)	2 (7%)	6 (7%)	0.97
Morbidity score	2 (1–3)	2 (1–3)	2 (1–3)	0.37
Dystonia	36 (60%)	17 (59%)	53 (60%)	0.90
Dystonia movement scale	1 (0–29.75)	0 (0–59.5)	0 (0–48)	0.58
Dystonia disability scale	0 (0–28)	0 (0–30)	0 (0–29)	0.37

**Fig. 1:**
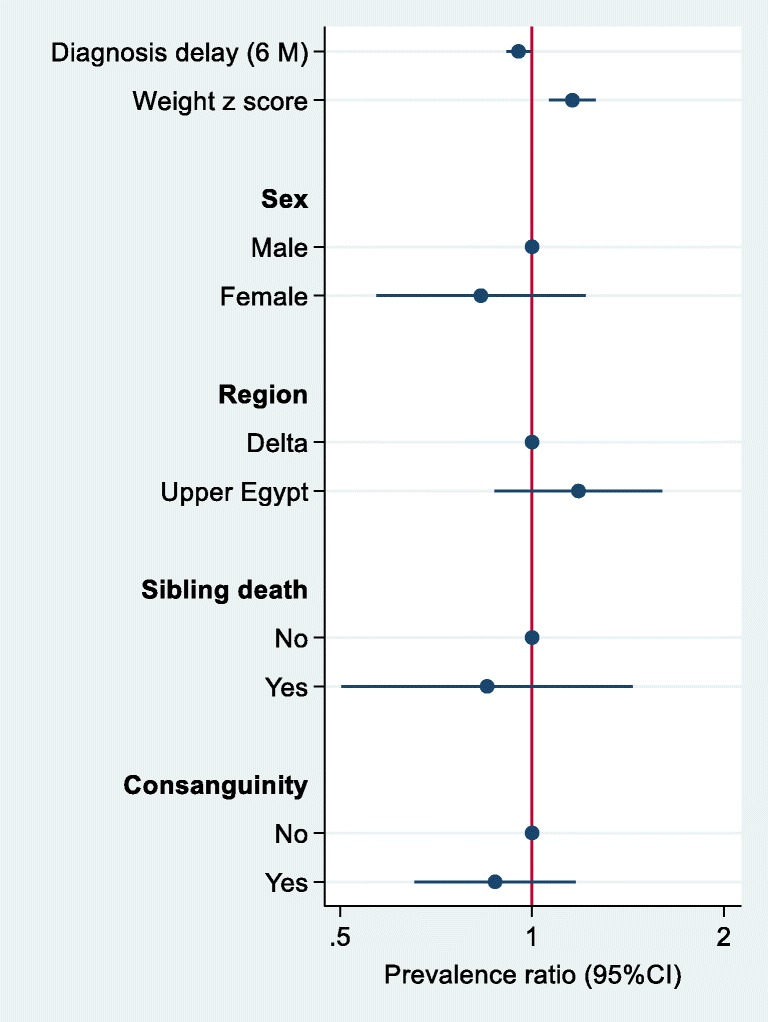
Prevalence rate ratio (95%CI) for macrocephaly by sample characteristics. The prevalence rate ratio was calculated using a Poisson regression with robust variance. The model adjusted for all variables in the fig. (*N* = 71)

**Fig. 2:**
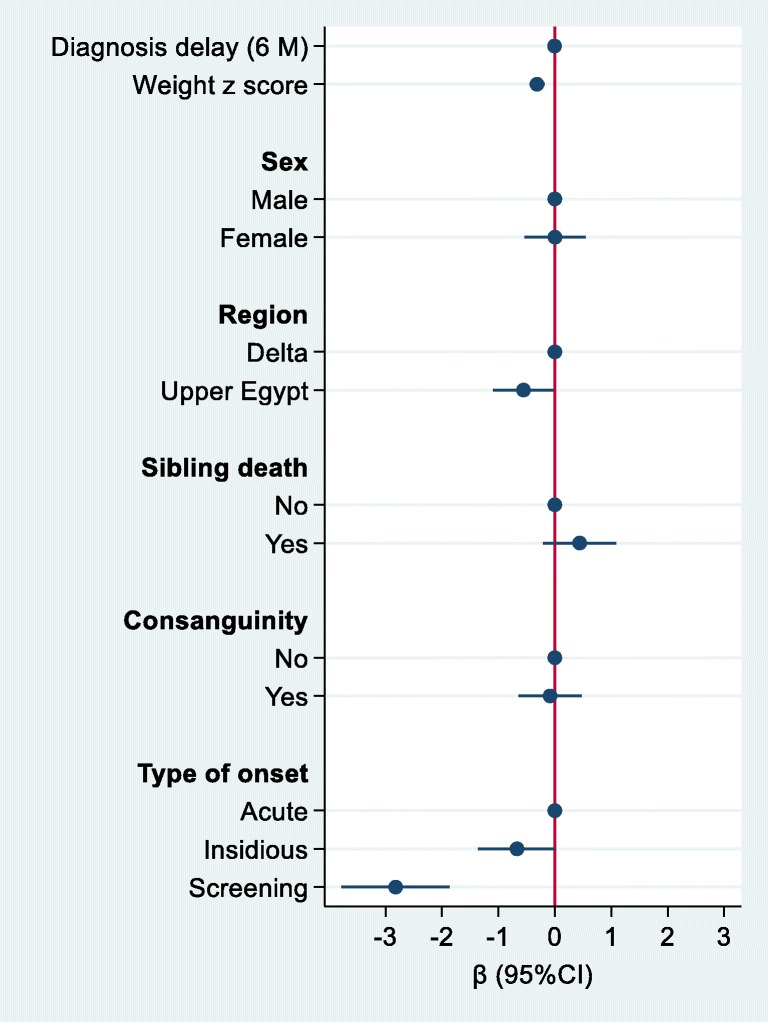
Regression coefficients (95%CI) for morbidity score by sample characteristics. The model adjusted for all the variables in the fig. (N = 71)

The severity and morbidity scores ranged from 0 to 4 according to the number of morbidities that were present (Supplementary Table 4). Most of the 89 patients demonstrated the acute-onset type (71.9%) followed by insidious (19%) and asymptomatic (9%), and convulsion manifested in 40.4% of cases with a median dystonia movement scale (IQR) of 48 and a range of 2–102. The dystonia disability scale showed a median of 29 and a range of 8–30. The morbidity score range was 0–4. Sixty-four (71.9%) of our patients were diagnosed with decompensation after an associated intercurrent infection. Eight (9.0%) of our asymptomatic patients were diagnosed by neonatal screening because of a positive family history or because of having another sibling that was diagnosed with GA1 (Table 1 and Supplementary Table 4). Patients who were diagnosed on screening had a much more favorable and significantly lower morbidity score compared to patients who were diagnosed later in life (Fig. 2). There was no severe cognition delay in the included children with GA1. Approximately 69% of our patients were diagnosed with macrocephaly with an occipitofrontal circumference (OFC) above the 97th percentile, while four (4.4%) of our patients were diagnosed with microcephaly with an OFC below the 3rd percentile (Supplementary Table 2). A delay in diagnosis was inversely associated with macrocephaly. The prevalence rate ratio (PR) for macrocephaly that was associated with each six-month delay was 0.95 (95%CI 0.91–0.99) (Fig. 1). However, a higher body weight was associated with a higher likelihood of having macrocephaly (PR 1.16, 95%CI 1.06–1.26 per 1 SD increment of weight Z score). However, body weight was inversely associated with morbidity score (Fig. 2). Patients who lived in Upper Egypt and the patients who were diagnosed by the screening of identified cases had a tendency to show lower morbidity scores.

**Fig. 3:**
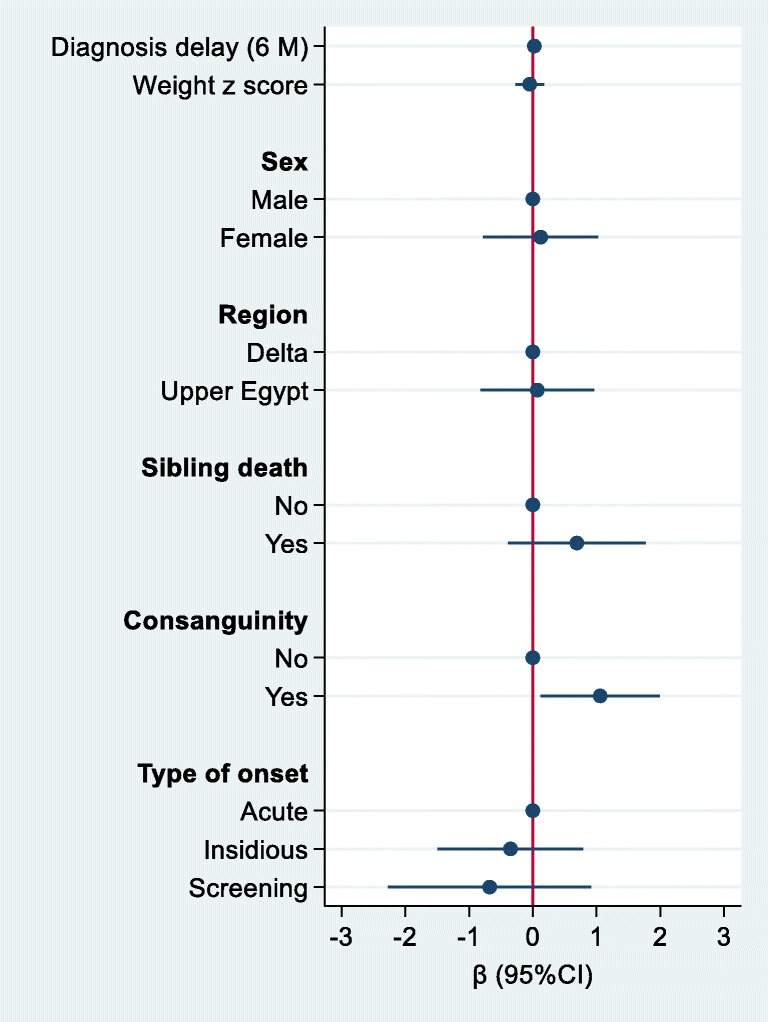
Regression coefficients (95%CI) for C5DC level by sample characteristics. The model adjusted for all the variables in the fig. (N = 71)

Biochemical analyses using MS/MS and GC/MS were performed for all patients and revealed that the C5DC level in blood was high and above the normal level of 0.3 μmol/L in all of our patients and ranged from 0.35–11.2 (median (IQR) 1.35 (0.83–2.17)). Most of the patients (93.3%) were high excretors (glutaric acid excretion of >100 mmol GA/mol creatinine, and only six (6.7%) were low excretors (<100 mmol GA/mol creatinine). High excretors, according to the urinary GC-MS measurements, were additionally divided into high (75.3%) and massive (18.0%) excretor groups (Supplementary Tables 6). Consanguinity was positively associated with the C5DC level (β (95%CI) 1.06 (0.12–1.99)) (Fig. 3).

## Molecular genetics analysis

Of the 89 patients, 41 were sequenced for the *GCDH* gene. The genotyping of the 41 patients identified 25 variants, including 14 missense variants, three frameshift, two nonsense, one silent, and five 3’-UTR variants. The most frequent missense variant is p.Arg402Trp, with 29 mutated alleles in 15 patients. The c.*165A > G variant was found in 21 patients in its homozygous form with an allele frequency of 0.512%. We identified six novel variants: three missense variants c.320G > T (p.Gly107Val), c.481C > T (p.Arg161Trp) and c.572 T > G (p.Met191Arg); two deletions c.78delG (p.Ala27Argfs34) and c.1035delG (p.Gly346Alafs*11); and one indel c.272_331del (p.Val91_Lys111delinsGlu); all variants were in homozygous form, and each of them was detected in a single patient with allele frequency of 0.0244 (Table 1). All of these variants were absent in 300 normal chromosomes of normal individuals of Arab descent. Functional analysis for these variants was not possible in our center.

## Computational analysis

We used six in silico prediction tools to predict the effect of the 14 missense variants on the GCDH protein; these prediction tools included three stability prediction tools (I-Mutant2.0, I-stable, and Mupro), two pathogenicity prediction tools (PolyPhen2 and SIFT), and one conservation analysis using the Consurf server. All missense variants were predicted as pathogenic by SIFT and polyphen2, except for the p.Ser139Leu, p.Arg128Gln, p.Glu64Asp, and p.Ala433Val variants, which were tolerated with SIFT (Table 3). The ConSurf server ranked the residues of GCDH according to their conservation with a gradient scale of 1–9, where a 1 score indicates a highly variable amino acid residue and a score of 9 indicates highly conserved residue. All amino acid positions at the 14 positions of the missense variants were highly conserved with scores of 8–9; however, four positions had an average conservation, with a score of 5 (Pro53, Trp50, Arg161, Arg161). The three stability predictors predicted that all of missense variants would decrease protein stability except for two variants (p.Ser119Leu p.Ser139Leu), which were predicted to increase the stability, and I-Mutant also predicated the p.Arg257Gln variant to increase the protein stability (Table 3).

## Discussion

In the present study, we aimed to characterize 89 patients who were diagnosed with GA1. Approximately 64% of our patients came from consanguineous families and 33.7% had a positive family history of the disease (Supplementary Table 1). The diagnosis was based on the clinical, neuroradiological, and biochemical characteristics of the patients. Over 93% of the patients demonstrated a high excretor phenotype in the urinary GC/MS organic acid analysis, indicating a deficient (≤5%) GCDH enzyme activity (Strauss et al. 2010). Approximately 60% percent of the patients in our cohort had dystonia and 40% suffered from convulsions, and these measurements are comparable with our earlier findings (Mosaeilhy et al. 2017). The neuroradiological examination demonstrated that the globus pallidus was the most affected area of the brain in 88.6% of the patients. This is consistent with our previous results of MRI scans of 29 children with GA1, where the globus pallidus was the most affected (86%) region among the 29 Egyptian patients (Mohammad et al. 2015). All of the patients presented with variable degrees of developmental delay ranging from mild to severe (Supplementary Tables 4–5). Twenty-five variants were identified in the 41 patients. Most of the variants that were identified were missense variants (65%, 14/25) (Table 2). We detected the following six novel variants: c.320G > T (p.Gly107Val), c.481C > T (p.Arg161Trp) and c.572 T > G (p.Met191Arg); the two deletions c.78delG (p.Ala27Argfs34) and c.1035delG (p.Gly346Alafs*11); and one indel c.272_331del (p.Val91_Lys111delinsGlu). All of the novel variants were absent in the 300 normal chromosomes. Five variants were detected in the 3’-UTR, of which the c.*165A > G variant was detected in 42 alleles (21 patients). The most detected missense variant c.1204C > T (p.Arg402Trp) was identified in 29 mutated alleles in 15/41 (~37%) of patients.

**Table 2:** Genetic analysis data of the 41 genetically sequenced patients with GA1

PN	SNP	N change	P change	Z	AN/82	C5DC Level (uMol/L)	Clinical Significance	Reference
P1	rs755586631	c.383G > A	p.Arg128Gln	H	2	2.39	LP	(Zschocke et al. 2000)
	rs1060218	c.1173G > T	p.Gly391=	H	8		B	
	rs8012	c.*165A > G	3`-UTR	H	42		NR	
	rs9384	c.*288G > T	3`-UTR	H	22		NR	
P2	rs121434369	c.1204C > T	P.Arg402Trp	H	29	1.33	LP	(Christensen et al. 2004)
P3	rs121434369	c.1204C > T	p.Arg402Trp	H	29	1.37	LP	(Christensen et al. 2004)
P4	rs121434369	c.1204C > T	p.Arg402Trp	H	29	4.48	LP	(Christensen et al. 2004)
P5	rs139851890	c.148 T > A	p.Trp50Arg	Ht	1	3.05	NA	(Mosaeilhy et al. 2017)
		c.416C > T	p.Ser139Leu	Ht	3		P/LP	
P6	rs121434369	c.1204C > T	P.Arg402Trp	H	29	2.06	LP	(Christensen et al. 2004)
P7	rs121434369	c.1204C > T	P.Arg402Trp	H	29	1.02	LP	(Christensen et al. 2004)
		c.*163 T > C	3`-UTR	H	4		NR	
P8	rs121434369	c.1204C > T	P.Arg402Trp	H	29	1.56	LP	(Christensen et al. 2004)
P9	rs139851890	c.416C > T	p.Ser139Leu	H	3	0.39	P/LP	(Goodman et al. 1998)
P10	–	c.1284C > G	p.Ile428Met	Het	1	1.32	NR	(Mosaeilhy et al. 2017)
	rs8012	c.*165A > G	3`-UTR	H	42		NR	
P11	rs121434369	c.1204C > T	P.Arg402Trp	H	29	2.24	LP	(Mosaeilhy et al. 2017)
P12	rs886043840	c.356C > T	p.Ser119Leu	H	2	1.42	CIP	(Korman et al. 2007)
P13	–	c.644_645insCTCG	p.Pro217Leufs*14	H	2	3.59	NA	(Moseilhy et al. 2016) (Mosaeilhy et al. 2017)
	–	c.*163 T > C	3`-UTR	H	4		NR	
	rs9384	c.*288G > T	3`-UTR	H	22		NR	
P14	rs751583656	c.770G > A	p.Arg257Gln	H	6	0.78	P	(Gupta et al. 2015)
	rs113720193	c.*161G > A	3`-UTR	H	18		NR	
	rs8012	c.*165A > G	3`-UTR	H	42		NR	
	rs9384	c.*288G > T	3`-UTR	H	22		NR	
P 15	rs1555749239	c.192G > T	p.Glu64Asp	Ht	5	0.63	LP	(Mosaeilhy et al. 2017)
	rs113720193	c.*161G > A	3`-UTR	H	18		NR	
	rs8012	c.*165A > G	3`-UTR	H	42		NR	
	rs9384	c.*288G > T	3`-UTR	H	22		NR	
P16	–	c.1189G > T	p.Glu397*	Ht	2	6.93	NR	(Mosaeilhy et al. 2017)
	rs113720193	c.*161G > A	3`-UTR	H	18		NR	
	rs8012	c.*165A > G	3`-UTR	H	42		NR	
	rs9384	c.*288G > T	3`-UTR	H	22		NR	
P17	rs121434369	c.1204C > T	P.Arg402Trp	Ht	29	1.88	LP	(Christensen et al. 2004)
	rs8012	c.*165A > G	3`-UTR	H	42		NR	
P18	rs751583656	c.770G > A	p.Arg257Gln	H	6	2.02	P	(Gupta et al. 2015)
P19	rs751583656	c.770G > A	p.Arg257Gln	H	6	2.49	P	(Gupta et al. 2015)
	rs113720193	c.*161G > A	3`-UTR	H	18		NR	
	rs8012	c.*165A > G	3`-UTR	H	42		NR	
	rs9384	c.*288G > T	3`-UTR	H	22		NR	
P20	–	c.158C > A	p.Pro53Gln	Het	3	11.2	NR	(Mosaeilhy et al. 2017)
	rs8012	c.*165A > G	3`-UTR	H	42		NR	
P21	rs113720193	c.*161G > A	3`-UTR	H	18	1.84	NR	(Mosaeilhy et al. 2017)
	rs8012	c.*165A > G	3`-UTR	H	42		NR	
P22	rs933624223	c.1298C > T	p.Ala433Val	H	2	1.44	VUS	(Busquets et al. 2000)
	rs8012	c.*165A > G	3`-UTR	H	42		NR	
P23	rs121434369	c.1204C > T	p.Arg402Trp	H	29	0.95	LP	(Christensen et al. 2004)
P24	–	c.572 T > G	p.Met191Arg	H	2	6.43	NA	**Novel**
	rs1060218	c.1173G > T	p.Gly391=	H	8		B	
	rs113720193	c.*161G > A	3`-UTR	H	18		NR	
	rs8012	c.*165A > G	3`-UTR	H	42		NR	
	rs9384	c.*288G > T	3`-UTR	H	22		NR	
P25	rs752334462	c.382 C > T	p.Arg128*	H	2	1.92	P	(Abdul Wahab et al. 2016)
	rs8012	c.*165A > G	3`-UTR	H	42		NR	
P26	rs777201305	c.482G > A	p.Arg161Gln	H	2	2.49	P/LP	(Busquets et al. 2000)
P27	rs121434369	c.1204C > T	p.Arg402Trp	H	29	1.22	LP	(Christensen et al. 2004)
	rs8012	c.*165A > G	3`-UTR	H	42		NR	
P28	–	c.158C > A	p.Pro53Gln	H	3	5.51	NA	(Mosaeilhy et al. 2017)
	rs8012	c.*165A > G	3`-UTR	H	42		NR	
P29	rs786204626	c.1205G > A	p.Arg402Gln	H	2	0.99	LP	(Christensen et al. 2004)
	rs8012	c.*165A > G	3`-UTR	H	42		NR	
P30	–	c.1035delG	p.Gly346Alafs*11	H	2	1.06	NA	**Novel**
	rs113720193	c.*161G > A	3`-UTR	H	18		NR	
	rs8012	c.*165A > G	3`-UTR	H	42		NR	
	rs9384	c.*288G > T	3`-UTR	H	22		NR	
P31	–	c.78delG	p.Ala27Argfs34	H	2	0.65	NA	**Novel**
	rs1060218	c.1173G > T	p.Gly391=	H	8		NR	
	rs8012	c.*165A > G	3`-UTR	H	42		NR	
P32	–	c.481C > T	p.Arg161Trp	H	2	2.05	NA	**Novel**
	rs8012	c.*165A > G	3`-UTR	H	42		NR	
P33	rs121434369	c.1204C > T	p.Arg402Trp	H	29	2.73	LP	(Christensen et al. 2004)
P34	rs121434369	c.1204C > T	p.Arg402Trp	H	29	0.63	LP	(Christensen et al. 2004)
P35	rs121434369	c.1204C > T	p.Arg402Trp	H	29	0.66	LP	(Christensen et al. 2004)
P36	–	c.320G > T	p.Gly107Val	H	2	1.76	NA	**Novel**
	rs113720193	c.*161G > A	3`-UTR	H	18		NR	
	rs8012	c.*165A > G	3`-UTR	H	42		NR	
	rs9384	c.*288G > T	3`-UTR	H	22		NR	
P37	rs121434369	c.1204C > T	p.Arg402Trp	H	29	3.17	LP	(Christensen et al. 2004)
P38	rs1555749239	c.192G > T	p.Glu64Asp	H	5	1.66	LP	(Christensen et al. 2004)
	rs113720193	c.*161G > A	3`-UTR		18		NR	
	rs8012	c.*165A > G	3`-UTR		42		NR	
	rs9384	c.*288G > T	3`-UTR		22		NR	
P39	rs121434369	c.1204C > T	p.Arg402Trp	H	29	1.02	LP	(Christensen et al. 2004)
	rs1060218	c.1173G > T	p.Gly391=		8		NR	
	rs8012	c.*165A > G	3`-UTR		42		NR	
	rs9384	c.*288G > T	3`-UTR		22		NR	
P40	rs1555749239	c.192G > T	p.Glu64Asp	H	5	2.17	LP	(Christensen et al. 2004)
P41	–	c.272_331del	p.Val91_Lys111delinsGlu	H	2	0.8	NA	**Novel**

Clinical significance was measured using the Clinvar databases. B = benign, LP = likely pathogenic, NA = not available (for novel variants), NR = not reported in Clinvar, H = homozygous, Ht = heterozygous, N = nucleotide, P = protein, AN = allele number. The ref.# used for nomenclature is NM_000159.3

The missense variant c.1204C > T (p.Arg402Trp) was identified in more than 35% of the alleles in 15 of our patients; of these, 57% of patients manifested with convulsions and 58.8% with macrocephaly. The C5DC, GA, and 3-OH-GA levels were abnormally elevated in all patients that carried this variant, and patients harboring this variant were all found to be high excretors (Supplementary Fig. 6). We previously detected this variant with an allele frequency of ~0.361 among 18 patients with GA1 (Mosaeilhy et al. 2017), and this variant is known to be common among Caucasian GA1 patients (Zschocke et al. 2000; Christensen et al. 2004). This finding is interesting for us, as the regions where the patients came from are very conservative regions in terms of marriage, where the prevalence of consanguineous marriage is very high (Mosaeilhy et al. 2017), thus suggesting a founder effect of this variant in this population. Cultured fibroblasts from homozygous patients with this variant demonstrated undetectable levels of GCDH enzyme activity (Christensen et al. 2004). In addition, in silico analyses have consistently predicted a deleterious effect of this variant (Table 3). All of these findings, together with the published data, suggest that this variant has a strong genotype-phenotype correlation; however, a recent report described that this variant was associated with a mild phenotype among Polish patients with GA1, and it was reported in 13/25 (52%) alleles among 13 patients (Pokora et al. 2019).

**Table 3:** In silico predictions for the pathogenicity of missense variants in our patients’ cohort

Variant	I-Mutant-2	Mupro	I-Stable	ConSurf	SIFT	Polyphen2	Reference
p.Met191Arg	D	D	D	9	NT	PD	This study
p.Arg161Trp	D	D	D	5	NT	PD	
p.Gly107Val	D	D	D	9	NT	PD	
p.Arg161Gln	D	D	D	5	NT	PD	(Busquets et al. 2000)
p.Ala433Val	D	D	D	8	T	PD	
p.Trp50Arg	D	D	D	5	NT	PD	(Mosaeilhy et al. 2017)
p.Pro53Gln	D	D	D	5	NT	PD	
p.Glu64Asp	D	D	D	9	T	PD	
p.Ser119Leu	I	I	I	9	NT	PD	
p.Arg128Gln	D	D	D	9	T	PD	
p.Ser139Leu	I	I	I	9	T	PD	
p.Arg257Gln	I	D	D	9	NT	PD	
p.Arg402Trp	D	D	D	9	NT	PD	
p.Ile428Met	D	D	D	8	NT	PD	

Of the six novel variants that we identified, the three missense mutations c.572 T > G (p.Met191Arg), c.481C > T (p.Arg161Trp), and c.320G > T (p.Gly107Val) were identified in patients 7, 32, and 36 in their homozygous form (Table 2). These patients presented with acute onset and severe dystonia and manifested with macrocephaly and convulsion (Supplementary Table 2 and 4). The biochemical analyses showed abnormally elevated levels of C5DC, GA and 3-OH-GA, and all patients are classified by their high excretor phenotype (Supplementary Table 6). In addition to these variants, patient 7 harbors three other variants (c.1173G > T (p.Gly391=), c.*161G > A, c.*165A > G, and c.*288G > T), patient 32 carries a 3`-UTR variant (c.*165A > G), and patient 36 harbors three 3`-UTR variants (c.*161G > A, c.*165A > G, and c.*288G > T) (Table 2). These three novel missense variants are predicted to decrease the stability of the protein, as determined with three protein stability predictors (I-Mutant-2, Mupro, I-Stable), and these variants were predicated as intolerated by SIFT and as probably damaging by Polyphen2 (Table 3). Both Met191 and Gly107 are highly conserved, and both were predicted to have the maximum conservation score of 9 by the Consurf server; however, Arg161 conservation shows an average conservation score of 5 (Table 3). The other three novel variants, the two deletions c.1035delG (p.Gly346Alafs*11) and c.78delG (p.Ala27Argfs34) and the one indel c.272_331del (p.Val91_Lys111delinsGlu), were detected in their homozygous form, and each of these variants were identified in patients 30, 31, and 41, respectively. These three patients showed high excretor phenotypes and had abnormally elevated levels of GA, 3-OH-GA, and C5DC (Supplementary Table 6). Patients 31 and 41 presented with macrocephaly; however, patient 30 did not. Patients 30 and 41 presented with an acute form of the disease; however, patient 31 presented with an insidious form. Patients 30 and 31 were positive for dystonia; however, patient 41 was negative. Although deletion variants that lead to frameshifts are expected to be severe variants, patients 30 and 31 presented with mild phenotypes that were related to fine motor, speech, and cognitive clinical manifestations, and the same phenomenon was observed in patient 41, who carried the p.Val91_Lys111delinsGlu variant (Supplementary Table 5). This could be due to the early crisis management practices and the highly educated parents who were very careful to provide adequate management during the days without a crisis to avoid the occurrence of additional crises. However, patient 41 developed a crisis at the time of writing this report, and this patient developed the full spectrum of the disease despite the meticulous care of parents and physicians.

The frequency of macrocephaly in this study is 69%, with an OFC above the 97th percentile. Four patients (4.4%) were diagnosed with microcephaly. The high frequency of macrocephaly in our patient cohort is noticeably high compared to that of our previous study (50%) (Mosaeilhy et al. 2017), and other ethnic groups such as the Japanese (31.6%) (Mushimoto et al. 2011); however, this frequency is comparable to Caucasians (65–75%) (Kolker et al. 2006; Kolker et al. 2007b). We found that patients with macrocephaly (69%) were diagnosed earlier than those without it (31%) (Supplementary Table 2), and this finding is consistent with a significant association between a delay in diagnosis and macrocephaly (Fig. 1). The prevalence rate ratio (PR) for macrocephaly that was associated with each six-month delay was 0.95 (95%CI 0.91–0.99) (Fig. 1); this can be explained because patients without macrocephaly would not be noticed at an earlier time point, and an early diagnosis could therefore be missed due to the wide variations in disease manifestations, consistent with our previous findings (Mosaeilhy et al. 2017).

Among our 89 patients, the median age of onset was 5.25 months, with a median six-month delay in diagnosis. This is comparable to our earlier published results of 18 Egyptian patients with GA1 where the median age at the onset of symptoms was six months, and the age at diagnosis was quite variable, with a gap of 12 months between onset age and diagnosis age (Mosaeilhy et al. 2017). In our patient cohort, our data appeared to show a negative correlation between the delay in diagnosis and age of onset (Supplementary Fig. 4). Thus, the earlier the manifestation started, the more likely it was to be missed, and patients with early onset usually retained neurological damage and commonly had the worst neurological prognoses (Bjugstad et al. 2000). Since the age at the onset of symptoms can significantly predict the severity of motor deficits and the overall outcome, it is important to identify patients with GA1 as early as possible, as presymptomatic treatment may prevent or postpone the onset of symptoms (Bjugstad et al. 2000), emphasizing the important of newborn screening. Nine of our patients were diagnosed by a neonatal screening due to their family history of the disease. These patients had a much better speech and fine and gross motor development and a significantly lower incidence of macrocephaly or muscular tone abnormalities than patients who were diagnosed by referral and were delayed in their diagnosis. The overall morbidity score of patients who were diagnosed at the neonatal screening was significantly more favorable compared to that of patients who were diagnosed later in life. This discrepancy might be attributed to the start of therapy before the onset of neurological complications (Lindner et al. 2004; Kolker et al. 2006); therefore, the early recognition of GA1 is thus the key to minimize GA1-associated morbidity, as the clinical presentation is often not specific before the onset of encephalopathic crises. Thus, neonatal screening for GCDH deficiency is a reliable method for the detection of presymptomatic patients, and it enables the early detection and preemptive management of affected newborns (Greenberg et al. 2002; Kolker et al. 2006).

We diagnosed more than 138 patients in our center over the past eight years; 89 of these patients were those who were described in this study, and 49 were previously described (Mohammad et al. 2015; Moseilhy et al. 2016; Mosaeilhy et al. 2017). Although there is an overlap between the clinical and genetic profile of Egyptian patients and patients from other ethnic groups, such as Japanese (Mushimoto et al. 2011), Indians (Gupta et al. 2015), and Caucasians (Goodman et al. 1998; Zschocke et al. 2000), the Egyptian patients appear to have distinct disease susceptibility genotypes that are responsible for GA1 phenotypes. This finding could be explained due to the significant history of admixing in Egypt between different ethnic groups, including Asians and Caucasians. However, there are large segments of the Egyptian population (Egypt consists of over 100 million citizens) that are very conservative in marriage that believe it is a shame to marry outside of the family, and most of our patient cohort came from this group. For example, the 18 patients we published earlier demonstrated 100% consanguinity that all came from Upper Egypt region (Mosaeilhy et al. 2017). In this study, consanguinity was significantly associated with C5DC levels (Fig. 3).

Our current study and our previous study (Mosaeilhy et al. 2017) indicate that phenotype could not be conclusively predicted from genotype in most Egyptian patients mainly due to the small number of patients and especially due to the novel variants. However, the c.1204C > T (p.Arg402Trp) variant, which had a detected frequency of over 35% in this study and 36% among the 18 patients of our previous study (Mosaeilhy et al. 2017), was classified by Clinvar as likely pathogenic (Table 2), and a meaningful genotype-phenotype relationship is therefore believed to exist for this variant.

Our study encountered some limitations. First, not all patients’ guardians provided a consent for the genetic study. Second, we were not able to understand the phasing of the identified variants due to the unavailability of parents’ samples in the study. Third, the low number of female patients. Finally, some of the clinical and radiological data were not available for all patients. Our center is the only metabolic diseases genetic unit in Egypt, which is situated in Cairo, so very few patients will be able to make this expensive journey to Cairo from different cities in Upper Egypt and Delta. Therefore, many patients could die due to delays in diagnoses (Mosaeilhy et al. 2017), lack of care, and poor economic conditions. Most of the patients are referred to our clinic from regions in Egypt where consanguinity is a cultural practice and where it is a shame to marry out of the family; therefore, awareness of the consequences of consanguineous marriage among families where the disease is prevalent is necessary to save lives. More importantly, the establishment of new genetic clinics across epidemic areas and the enhancement of neonatal screening are necessary practices to avoid further loss of life. Therefore, our results are important in providing genetic and clinical counseling to Egyptian patients with GA1 and could serve as a platform for the prenatal diagnosis of GA1 in Egypt. Some of the problems that medical geneticists must be aware of include the delay of diagnosis, the long distance to the metabolic clinic, and the lack of awareness among patients and physicians. Since very few physicians in Egypt are specialized in medical genetics, it is important to bring the attention of all of the pediatricians in Egypt to such a devastating disease. General and emergency care pediatricians should consider GA1 in parallel with infectious and vascular causes in a previously healthy infant who presents with acute encephalitis or stroke-like illness. Neurosurgeons and other medical staff who evaluate patients with head trauma or suspected non-accidental head injury should include GA1 in the differential diagnosis of extracerebral fluid or blood collection. The combination of these factors leads us to believe that this disease is uncommon in Egypt and must be included in the comprehensive newborn screening practices in Egypt, which lacks a comprehensive program. Applying such a program will allow for the timely intervention of treatment and diet and will potentially prevent further neurological damage.

## Electronic supplementary material

ESM 1 (PDF 317 kb)
