# Supplementary material for: Clinical, biochemical, neuroradiological and molecular characterization of Egyptian patients with glutaric acidemia type 1
Source: Metab Brain Dis. 2019 May 6;34(4):1231–41. doi: 10.1007/s11011-019-00422-3 (PMC6617250; doi:10.1007/s11011-019-00422-3)
Supplement: Supplementary file 1 — (PDF 317 kb) [file 11011_2019_422_MOESM1_ESM.pdf]

Clinical, biochemical, neuroradiological and molecular characterization of Egyptian patients with glutaric acidemia type 1

Hatem Zayed[1], Hamed El Khayat[2], Hoda Tomoum[2], Ola Khalifa[2], Ehab Siddiq[2], Shaimaa A Mohammad[2], Radwa Gamal[2], Zumin Shi[1], Ahmed Mosailhy[2], Osama K Zaki[2]

1. *College of Health Sciences, Biomedical Program, Qatar University, Doha, Qatar*

2. *Medical Genetics Unit, Pediatric Department, Faculty of Medicine, Ain-Shams University, Cairo, Egypt.*

*__Correspondence to:__

Hatem Zayed, Ph.D., Department of Health Sciences, Biomedical Program, Qatar University, Doha, Qatar, hatem.zayed@qu.edu.qa

Phone: 00974-4403-4809

Fax: 00974-4403-1351

Osama K Zaki, M.D., Ph.D., Genetics Unit, Ain Shams Pediatrics Hospital, Cairo, 11665 ozaki@med.asu.edu.eg

Phone: +20105188879

Fax: 202 27712258

Supplementary Table 1. Clinical data of the Egyptian patients with GA1

PATIENT N.	AGE (MON)	SEX	RESIDENCE	FAMILY HISTORY	CONSANGUINITY
1.	40.00	F	U	-VE	+VE
2.	33.00	M	L	-VE	+VE
3.	50.00	M	L	-VE	-VE
4.	89.00	F	L	-VE	+VE
5.	92.00	M	U	+VE	+VE
6.	73.00	M	U	+VE	+VE
7.	61.00	F	U	+VE	+VE
8.	92.00	F	L	-VE	+VE
9.	90.00	M	L	+VE	-VE
10.	70.00	M	L	+VE	-VE
11.	89.00	M	L	-VE	+VE
12.	87.00	M	U	+VE	+VE
13.	28.00	F	U	+VE	+VE
14.	104.00	F	U	+VE	-VE
15.	72.00	M	U	+VE	-VE
16.	64.00	M	U	+VE	+VE
17.	49.00	M	U	+VE	+VE
18.	81.00	M	L	-VE	+VE
19.	85.00	M	L	+VE	+VE
20.	82.00	F	U	+VE	+VE
21.	134.00	M	U	+VE	+VE
22.	53.00	M	U	+VE	+VE
23.	34.00	M	U	+VE	+VE
24.	103.00	M	L	+VE	+VE
25.	70.00	M	U	-VE	+VE
26.	31.00	F	U	-VE	+VE
27.	33.00	M	U	-VE	-VE
28.	98.00	M	L	+VE	+VE
29.	64.00	M	L	+VE	+VE
30.	57.00	F	U	-VE	+VE
31.	36.00	M	L	-VE	+VE
32.	36.00	M	L	-VE	+VE
33.	103.00	F	L	-VE	+VE
34.	106.00	M	U	-VE	+VE
35.	141.00	F	L	-VE	-VE
36.	70.00	F	L	-VE	+VE
37.	69.00	M	U	-VE	+VE
38.	66.00	F	L	-VE	+VE
39.		M	L	-VE	-VE
40.	59.00	M	U	-VE	-VE
41.	50.00	F	L	-VE	+VE
42.	76.00	M	L	-VE	-VE
43.	59.00	M	U	-VE	+VE
44.	82.00	M	L	-VE	+VE
45.	80.00	F	U	-VE	-VE
46.	99.00	F	U	-VE	-VE
47.	54.00	M	U	-VE	+VE
48.	120.00	F	L	+VE	-VE
49.	85.00	M	L	+VE	-VE
50.	108.00	M	L	-VE	+VE

51.	140.00	F	L	+VE	+VE
52.	105.00	F	L	+VE	+VE
53.	95.00	F	U	-VE	-VE
54.	87.00	F	U	-VE	+VE
55.	104.00	F	L	-VE	+VE
56.	107.00	M	L	-VE	+VE
57.	215.00	M	U	-VE	-VE
58.	107.00	M	L	-VE	+VE
59.	149.00	F	L	-VE	-VE
60.	111.00	M	L	-VE	-VE
61.	88.00	M	U	-VE	+VE
62.	83.00	M	L	-VE	-VE
63.	252.00	M	U	-VE	-VE
64.	132.00	M	L	-VE	-VE
65.	71.00	F	L	+VE	+VE
66.	54.00	M	L	+VE	+VE
67.	87.00	M	L		+VE
68.		M	U	+VE	+VE
69.		M	U	+VE	+VE
70.	88.00	M	U	+VE	+VE
71.	100.00	M	U	+VE	+VE
72.	98.00	M	U	-VE	-VE
73.		M	U	-VE	-VE
74.	100.00	M	U	-VE	-VE
75.	59.00	M	U	-VE	-VE
76.	55.00	M	L	-VE	-VE
77.		F	U	-VE	-VE
78.		M	U	-VE	-VE
79.		F	L	-VE	-VE
80.		M	L	-VE	+VE
81.		F	L	-VE	-VE
82.	187.00	M	U	-VE	+VE
83.	50.00	F	U	-VE	+VE
84.		M	L	-VE	+VE
85.	36.00	M	U	-VE	+VE
86.	88.00	M	U	-VE	+VE
87.		M	U	-VE	-VE
88.	30.00	F	U	-VE	-VE
89.	69.00	M	L	-VE	+VE

Supplementary Table 2. Clinical data of Egyptian patients with GA1

PATIENT N.	OFC CENTILE AT DIAGNOSIS	OFC (Z. SCORE)	MACROCEPHALY	W.T CENTILE AT DIAGNOSIS	W.T (Z. SCORE)	LENGTH CENTILE AT DIAGNOSIS	LENGTH (Z. SCORE)
1.	25th	-0.662	-VE	7th	1.465	72 nd	0.577
2.	>99	3.612	+VE	30th	-0.512	60th	0.233
3.	75	0.594	-VE	60th	0.267	20th	-0.857
4.	>99	2.431	+VE	6th	-1.596	< 3rd	-2.629
5.	76th	0,709	-VE	4th	-1.775	21th	-0.826
6.	>99	3.796	+VE	30th	-0.516	61th	0.279
7.	65th	0.377	-VE	40th	-0.287	60th	0.238
8.	6th	-1.572	-VE	8th	-1.41	< 3rd	-2.742
9.	98th	1.983	+VE	67th	0.45	61th	0.275
10.	>99	3.467	+VE	40th	-0.287	60th	0.234
11.	>99	3.836	+VE	95th	1.617	99th	2.21
12.	18th	-0.919	-VE	< 3rd	-3.669	< 3rd	-4.948
13.	< 3rd	-3.48	-VE	< 3rd	-3.298	< 3rd	-2.845
14.	>99	3.675	+VE	40th	-0.282	60th	0.241
15.	>99	3.554	+VE	40th	-0.280	60th	0.232
16.	>99	2.693	+VE	< 3rd	-3.663	< 3rd	-6.251
17.	65th	0.375	-VE	87th	1.14	3rd	-1.913
18.	99th	6.697	+VE	92nd	1.397	< 3rd	-2.853
19.	>99	4.65	+VE	13th	-1.127	97th	1.823
20.	99th	2.279	+VE	91th	1.358	99th	6.014
21.	>99	3.323	+VE	40th	-0.287	60th	0.232
22.	>99	4.65	+VE	< 3 rd	-2.505	< 3 rd	-2.627
23.	>99	2.934	+VE	40th	-0.281	60th	0.237
24.	99th	2.601	+VE	6th	-1.52	< 3rd	-2.988
25.	99th	2.539	+VE	34th	-0.409	86th	1.059
26.	>99	3.905	+VE	30th	-0.512	6th	-1.56
27.	>99	3.058	+VE	89th	1.206	91st	1.368
28.	50th	-0.008	-VE	< 3rd	-2.472	20th	-0.857
29.	79th	0.803	-VE	40th	-0.287	60th	0.243
30.	32nd	-0.48	-VE	7th	-1.516	71st	0.544
31.	99th	2.747	+ve	30th	-0.53	59th	0.231
32.	>99	3.504	+VE	40th	-0.286	60th	0.243
33.	99th	2.185	+ve	< 3rd	-2.653	75th	0.643
34.	>99	3.031	+ve	66th	0.401	20th	-0.857
35.	>99	3.411	+VE	40th	-0.287	60th	0.241
36.	< 3rd	-2.663	-VE	< 3rd	-3.054	< 3rd	-2.011
37.	>99	2.669	+ve	5th	-5.052	60th	0.240
38.	92nd	2.103	-VE	40th	-0.286	60th	0.243
39.	>99	3.412	+VE	40th	-0.284	60th	0.245
40.	>99	3.745	+VE	40th	-0.289	60th	0.243
41.	99th	2.401	+VE	< 3rd	-2.749	45th	-0.127
42.	>99	3.792	+VE	40th	-0.282	60th	0.247
43.	>95	2.47	-VE	97th	2.494	47th	-0.07
44.	>99	2.669	+VE	26th	-0.635	23rd	-0.736
45.	>99	3.255	+VE	11th	-1.218	81st	0.859
46.	>99	3.404	+VE	16th	-0.99	95th	1.66
47.	>99	4.835	+VE	16th	-1.01	38th	-0.313
48.	99th	4.597	+VE	21th	-0.807	39th	-0.276
49.	99th	4.889	+VE	71th	0.558	99th	2.776

50.	9th	-1.381	-VE	< 3rd	-5.079	< 3rd	-5.688
51.	5th	-1.802	-VE	< 3rd	-3.994	60th	0.240
52.	43d	-0.172	-VE	< 3rd	-2.226	60th	0.293
53.	84th	0.975	-VE	57th	0.167	97th	1.822
54.	>99th	2.669	+ve	98th	1.994	60th	0.244
55.	99th	3.49	+VE	20th	-0.759	99th	2.223
56.	98th	1.983	+VE	98th	3.915	98th	3.914
57.	< 3rd	-2.8	-VE	5th	-7.926	5th	-4.753
58.	98tth	2.119	+VE	42nd	-0.196	60th	0.238
59.	< 3rd	-3.96	-VE	< 3rd	-2.581	< 3rd	-4.092
60.	6th	-1.541	-VE	< 3rd	-3.716	3rd	-3.746
61.	35th	-0.423	-VE	< 3rd	-2.551	40th	-0.269
62.	98th	2.147	+VE	9th	-1.481	9th	-1.517
63.	10th	-1.333	-VE	5th	-4.939	5th	-6.021
64.	40th	-0.253	-VE	10th	-1.278	< 3rd	-4.396
65.	99th	2.43	+VE	57th	0.167	20th	-1.281
66.	69th	0.494	-VE	40th	-0.187	60th	0.243
67.	27th	-0.621	-VE	3rd	-1.892	2nd	-2.574
68.	99th	2.248	+VE	< 3rd	-3.832	21st	-0.826
69.	>99	3.404	+VE	40th	-0.287	60th	0.243
70.	>99	3.417	+VE	40th	-0.287	60th	0.235
71.	99th	3.773	+VE	40th	-0.287	60th	0.248
72.	99th	2.28	+VE	63th	0.328	60th	0.244
73.	>99	3.356	+VE	45th	-0.123	60th	0.234
74.	>99	3.478	+VE	45th	-0.122	60th	0.237
75.	>99	3.564	+VE	45th	-0.121	60th	0.245
76.	99th	2.389	+VE	54th	0.101	60th	0.241
77.	>99	3.452	+VE	45th	-0.126	60th	0.243
78.	>99	3.884	+VE	45th	-0.127	60th	0.236
79.	>99	3.477	+VE	45th	-0.129	23rd	-0.737
80.	96th	1.756	-VE	45th	-0.121	60th	0.240
81.	31st	-0.501	-VE	< 3rd	-5.497	60th	0.241
82.	>99th	3.151	+VE	99th	5.309	99th	5.367
83.	>99	3.411	+VE	45th	-0.126	60th	0.242
84.	98th	2.045	+VE	70th	0.488	80th	0.843
85.	97th	1.777	+VE	45th	-0.129	70th	0.562
86.	98th	2.147	+VE	13th	-1.147	99th	2.776
87.	>99	3.488	+VE	45th	-0.127	70th	0.567
88.	>99	3.932	+VE	45th	-0.122	70th	0.569
89.	>99	3.473	+VE	45th	-0.120	70th	0.563

Supplementary Table 3. Clinical data of the Egyptian patients with GA1

PATIENT N	DYSTONIA	DYSTONIA MOVEMENT SCALE	DYSTONIA DISABILITY SCALE	ABORTIONS	SIB DEATH
1.	-VE	0	0	0	+VE
2.	-VE	0	0	0	-VE
3.	-VE	0	0	0	-VE
4.	-VE	0	0	3	-VE
5.	+VE	52.5	28	1	+VE
6.	-VE	0	0	1	+VE
7.	+VE			1	+VE

8.	-VE	0	0	0	-VE
9.	-VE	0	0	1	+VE
10.	+VE	4	28	0	-VE
11.	+VE	48	30	1	-VE
12.	-VE	0	0	1	+VE
13.	+VE	70	30	2	+VE
14.	-VE	0	0	0	-VE
15.	+VE	59.5	29	0	-VE
16.	-VE	0	0	0	+VE
17.	+VE	29.5	30	0	+VE
18.	-VE	0	0	0	+VE
19.	+VE	102	29	0	+VE
20.	+VE	50.5	30	1	-VE
21.	+VE			0	-VE
22.	+VE	3	17	0	-VE
23.	+VE			0	-VE
24.	+VE			0	+VE
25.	-VE	0	0	0	-VE
26.	+VE			0	-VE
27.	+VE			0	-VE
28.	+VE	30	0	0	-VE
29.	+VE			0	-VE
30.	+VE	2	8	0	-VE
31.	+VE	10.5	0	0	-VE
32.	+VE	12	26	0	-VE
33.	-VE	0	0	1	+VE
34.	-VE	0	0	1	-VE
35.	-VE	0	0	0	-VE
36.	-VE	0	0	1	-VE
37.	+VE	52.5	28	0	-VE
38.	-VE	0	0	0	-VE
39.	+VE			0	-VE
40.	-VE	0	0	0	-VE
41.	-VE	0	0	1	-VE
42.	+VE	4	28	0	-VE
43.	+VE	48	30	0	-VE
44.	-VE	0	0	0	-VE
45.	+VE	70	30	0	-VE
46.	-VE	0	0	0	-VE
47.	+VE	59.5	29	0	-VE
48.	-VE	0	0	0	-VE
49.	+VE	29.5	30	0	-VE
50.	-VE	0	0	0	+VE
51.	+VE	102	29	0	-VE
52.	+VE	50.5	30	0	-VE
53.	+VE			0	-VE
54.	+VE	3	17	0	-VE
55.	+VE			0	-VE
56.	+VE			0	-VE
57.	-VE	0	0	0	-VE
58.	+VE			0	+VE
59.	+VE			0	-VE
60.	+VE	30	0	0	-VE

61.	+VE			1	-VE
62.	+VE	2	8	0	-VE
63.	+VE	10.5	0	1	-VE
64.	+VE	12	26	0	-VE
65.	-VE	0	0	0	-VE
66.	-VE	0	0	0	-VE
67.	-VE	0	0	2	-VE
68.	-VE	0	0	0	-VE
69.	+VE	52.5	28	0	-VE
70.	-VE	0	0	0	-VE
71.	+VE			0	-VE
72.	-VE	0	0	0	-VE
73.	-VE	0	0	0	-VE
74.	+VE	4	28	0	-VE
75.	+VE	48	30	0	-VE
76.	-VE	0	0	1	-VE
77.	+VE	70	30	0	-VE
78.	-VE	0	0	0	-VE
79.	+VE	59.5	29	0	-VE
80.	-VE	0	0	1	-VE
81.	+VE	29.5	30	0	+VE
82.	-VE	0	0	0	-VE
83.	+VE	102	29	0	-VE
84.	+VE	50.5	30	1	-VE
85.	+VE			2	-VE
86.	+VE	3	17	0	-VE
87.	+VE			0	-VE
88.	+VE			0	-VE
89.	-VE	0	0	0	-VE

Supplementary Figure 4. Clinical data of Egyptian patients with GA1

Patient N.	Age of onset(M)	Delay in diagnosis(M)	Type of onset	Onset-Associated intercurrent infection	Intra Cranial Hge	Convulsions	Morbidity Score	Fate& severity
1.	5	7.00	acute	+VE	-VE	-VE	1	mild
2.			acute	+VE	-VE	+VE	2	moderate
3.			acute	+VE	-VE	+VE	2	moderate
4.	3	6.00	acute	+VE	-VE	+VE	4	Severe
5.	7	7.00	acute	+VE	+VE	-VE	3	Severe
6.	2.7	0.30	insidious	-VE	-VE	+VE	3	Severe
7.			acute	+VE	-VE	+VE	4	Severe
8.	6	9.00	Screening	-VE	-VE	-VE	0	mild

9.	12	6.00	acute	+VE	-VE	-VE	3	Severe
10.		0.00	Screening	-VE	-VE	-VE	0	mild
11.	6	6.00	acute	+VE	-VE	-VE	0	mild
12.	0	24.00	acute	+VE	-VE	+VE	4	Severe
13.	0.17	0.00	insidious	-VE	-VE	-VE	2	moderate
14.		0.00	acute	+VE	-VE	-VE	0	mild
15.		0.00	insidious	-VE	-VE	-VE	3	Severe
16.	3	3.00	acute	+VE	+VE	+VE	4	Severe
17.	0	0.17	insidious	-VE	-VE	-VE	1	mild
18.	2	11.00	insidious	-VE	-VE	-VE	0	mild
19.	4	2.00	insidious	-VE	-VE	-VE	3	severe
20.	0	12.00	acute	+VE	-VE	-VE	0	mild
21.	0	72.00	insidious	-VE	-VE	-VE	0	mild
22.	1.5	4.50	acute	+VE	-VE	+VE	2	moderate
23.			acute	+VE	-VE	-VE	0	mild
24.	10	9.00	acute	+VE	-VE	-VE	3	severe
25.	9	2.00	acute	+VE	-VE	-VE	1	mild
26.	11	9.00	acute	+VE	-VE	+VE	2	moderate
27.	6	6.00	acute	+VE	-VE	-VE	2	moderate
28.	6	10.00	insidious	-VE	-VE	-VE	3	severe
29.	1	0.00	Screening	-VE	-VE	-VE	0	mild
30.	14	1.00	acute	+VE	-VE	+VE	3	severe
31.			insidious	-VE	-VE	-VE	1	mild
32.			acute	+VE	+VE	+VE	3	severe
33.	0	6.00	acute	+VE	-VE	+VE	4	severe
34.	12	4.00	acute	+VE	-VE	+VE	2	moderate
35.		0.00	insidious	-VE	-VE	-VE	3	severe
36.	7	8.00	acute	+VE	+VE	+VE	4	severe
37.	0	12.00	acute	+VE	-VE	+VE	4	Severe
38.	5.5	0.50	acute	+VE	-VE	-VE	3	severe
39.		0.00	acute	+VE	-VE	-VE	1	mild
40.		0.00	acute	+VE	-VE	+VE	2	moderate
41.	0	7.00	acute	+VE	-VE	-VE	1	mild
42.			acute	+VE	-VE	-VE	2	moderate
43.			acute	+VE	+VE	-VE	3	severe
44.			Screening	-VE	-VE	-VE	1	mild
45.			acute	+VE	-VE	+VE	3	severe
46.	6	60.00	acute	+VE	-VE	+VE	4	Severe
47.			acute	+VE	-VE	-VE	2	moderate
48.	9	27.00	acute	+VE	-VE	+VE	4	severe
49.	4	7.00	acute	+VE	-VE	-VE	1	mild
50.	14	22.00	acute	+VE	-VE	+VE	4	severe
51.	7	39.00	acute	+VE	-VE	+VE	4	Severe
52.	3	7.00	Screening	-VE	-VE	-VE	0	mild
53.	10	5.00	acute	+VE	-VE	+VE	3	severe
54.	7	5.00	acute	+VE	-VE	+VE	2	moderate
55.	0	8.00	insidious	-VE	-VE	-VE	2	moderate
56.	2	6.00	acute	+VE	-VE	+VE	4	severe
57.	1	119.00	insidious	-VE	-VE	+VE	3	severe
58.	6	11.00	acute	+VE	-VE	+VE	4	severe
59.	12	49.00	acute	+VE	-VE	+VE	3	severe
60.	14	13.00	acute	+VE	-VE	+VE	4	severe
61.	7	1.00	acute	+VE	-VE	+VE	3	severe
62.	6	5.00	acute	+VE	-VE	+VE	4	severe
63.	0	177.00	insidious	-VE	-VE	+VE	3	severe
64.	7	14.00	acute	+VE	-VE	+VE	4	Severe
65.	15	0.00	acute	+ve	-VE	-VE	1	mild
66.	0	0.33	Screening	-VE	-VE	-VE	0	mild
67.	0	35.00	insidious	-VE	-VE	-VE	2	moderate

68.	5	9.00	acute	+VE	-VE	+VE	4	Severe
69.			Screening	-VE	-VE	-VE	0	mild
70.	18	8.00	acute	+VE	-VE	-VE	2	moderate
71.	0	38.00	insidious	-VE	-VE	-VE	0	mild
72.	7	5.00	acute	+VE	-VE	+VE	2	moderate
73.		0.00	acute	+VE	-VE	-VE	0	mild
74.		0.00	acute	+VE	-VE	-VE	1	mild
75.		0.00	acute	+VE	-VE	-VE	2	moderate
76.	0	9.00	acute	+VE	-VE	-VE	1	mild
77.			acute	+VE	-VE	-VE	2	moderate
78.			acute	+VE	-VE	-VE	2	moderate
79.	6	2.00	acute	+VE	-VE	-VE	2	moderate
80.			acute	+VE	-VE	-VE	1	mild
81.	0	84.00	Screening	-VE	-VE	-VE	1	mild
82.	0	72.00	insidious	-VE	-VE	-VE	0	mild
83.	5	12.00	acute	+VE	+VE	-VE	2	moderate
84.	6	1.00	acute	+VE	-VE	+VE	2	moderate
85.	0	8.50	insidious	-VE	-VE	-VE	1	mild
86.	7	4.00	acute	+VE	-VE	+VE	2	moderate
87.			acute	+VE	-VE	-VE	1	mild
88.			acute	+VE	-VE	-VE	1	mild
89.			acute	+VE	-VE	-VE	1	mild

Supplementary Figure 5. Neuroradiological data of the Egyptian patients with GA1

Patient N.	Tone	Gross motor	Fine Motor	Speech	Cognitive
1.	N	mild	mild	mild	mild
2.	hyper	severe	moderate	moderate	mild
3.	hyper	severe	moderate	moderate	mild
4.	hypo	severe	severe	severe	moderate
5.	hyper	severe	severe	severe	mild
6.	hyper	severe	moderate	moderate	mild
7.	hyper	moderate	moderate	moderate	mild
8.	N	mild	mild	mild	mild
9.	hyper	severe	severe	severe	moderate
10.	N	mild	mild	mild	mild
11.	N	mild	mild	mild	mild
12.	hyper	severe	severe	severe	mild
13.	hypo	moderate	moderate	moderate	moderate
14.	N	mild	mild	mild	mild
15.	hyper	severe	severe	severe	mild
16.	hyper	severe	severe	severe	mild
17.	hypo	moderate	moderate	moderate	mild
18.	hypo	severe	severe	severe	mild
19.	hyper	severe	moderate	moderate	mild
20.	hypo	moderate	mild	mild	mild
21.	hypo	mild	moderate	mild	mild
22.	hypo	moderate	moderate	moderate	moderate
23.	N	mild	mild	mild	mild
24.	hyper	severe	severe	severe	mild
25.	N	mild	mild	mild	mild
26.	hyper	severe	severe	moderate	moderate
27.	hyper	severe	moderate	severe	mild
28.	hyper	severe	moderate	mild	mild
29.	hypo	mild	moderate	moderate	mild

30.	hyper	severe	moderate	moderate	mild
31.	hypo	moderate	moderate	moderate	mild
32.	hyper	severe	moderate	moderate	mild
33.	hyper	severe	severe	severe	mild
34.	hyper	severe	moderate	moderate	moderate
35.	hyper	severe	moderate	moderate	mild
36.	hyper	severe	moderate	moderate	moderate
37.	hyper	severe	severe	severe	mild
38.	hyper	severe	severe	severe	mild
39.	N	mild	mild	mild	mild
40.	hyper	severe	moderate	severe	mild
41.	hypo	severe	moderate	moderate	mild
42.	hyper	severe	moderate	moderate	mild
43.	hyper	severe	severe	moderate	mild
44.	hypo	severe	severe	moderate	mild
45.	hyper	severe	severe	severe	mild
46.	hyper	severe	moderate	severe	mild
47.	hyper	severe	severe	severe	moderate
48.	hyper	severe	severe	moderate	mild
49.	hypo	mild	moderate	mild	mild
50.	hyper	severe	moderate	moderate	moderate
51.	hyper	severe	severe	severe	mild
52.	N	mild	mild	mild	mild
53.	hyper	severe	moderate	moderate	mild
54.	hyper	severe	moderate	moderate	moderate
55.	hyper	severe	moderate	moderate	mild
56.	hyper	severe	severe	moderate	mild
57.	hyper	severe	moderate	moderate	mild
58.	hyper	severe	severe	severe	mild
59.	hyper	severe	severe	severe	moderate
60.	hyper	severe	severe	severe	moderate
61.	hyper	severe	severe	severe	mild
62.	hyper	severe	severe	severe	mild
63.	hyper	moderate	moderate	moderate	mild
64.	hyper	moderate	moderate	moderate	mild
65.	hypo	moderate	mild	mild	mild
66.	hypo	moderate	moderate	mild	mild
67.	hyper	severe	severe	severe	mild
68.	hyper	severe	severe	severe	mild
69.	hypo	moderate	moderate	moderate	mild
70.	hyper	severe	moderate	moderate	mild
71.	hypo	mild	mild	moderte	mild
72.	hyper	severe	moderate	severe	mild
73.	hypo	moderate	severe	severe	mild
74.	hypo	moderate	moderate	severe	mild
75.	hyper	severe	severe	severe	mild
76.	hypo	moderate	severe	moderate	moderate
77.	hyper	severe	severe	moderate	mild
78.	hyper	severe	moderate	moderate	mild
79.	hyper	severe	moderate	severe	mild
80.	hypo	moderate	moderate	moderate	moderate
81.	hypo	moderate	moderate	moderate	mild
82.	hypo	moderate	moderate	moderate	mild
83.	hyper	severe	moderate	moderate	mild
84.	hyper	severe	moderate	moderate	mild
85.	hypo	moderate	moderate	mild	mild
86.	hyper	severe	moderate	severe	mild
87.	hypo	moderate	severe	moderate	mild
88.	hypo	moderate	severe	mild	mild

89.	hypo	moderate	severe	moderate	mild

Supplementary Table 6. Biochemical data of the Egyptian patients with GA1

Patient	C5DC Level (uMol/L)	GC-MS(GA and 3-OH-GA)	Excretor
1.	2.39	Massive	high excretor
2.	1,33	Highly	high excretor
3.	1.37	Highly	high excretor
4.	4.48	Massive	high excretor
5.	3.05	Massive	high excretor
6.	2.06	Highly	high excretor
7.	1.02	Highly	high excretor
8.	1.56	Highly	high excretor
9.	0.39	Small	low excretor
10.	1.32	Highly	high excretor
11.	2.24	Highly	high excretor
12.	1.42	Highly	high excretor
13.	3.59	Massive	high excretor
14.	0.78	Highly	high excretor
15.	0.63	Highly	high excretor
16.	6.93	Massive	high excretor
17.	1.88	Highly	high excretor
18.	2.02	Highly	high excretor
19.	2.49	Highly	high excretor
20.	11.2	Highly	high excretor
21.	1.84	Highly	high excretor
22.	1.44	Highly	high excretor
23.	0,95	Highly	high excretor
24.	6.43	Massive	high excretor
25.	1.92	Highly	high excretor
26.	2.49	Massive	high excretor
27.	1.22	Highly	high excretor
28.	5.51	Massive	high excretor
29.	0.99	Highly	high excretor
30.	1.06	Highly	high excretor
31.	0.65	Highly	high excretor
32.	2.05	Highly	high excretor
33.	2.73	Highly	high excretor
34.	0.63	Highly	high excretor
35.	0.66	Highly	high excretor
36.	1.76	Highly	high excretor
37.	3.17	Massive	high excretor
38.	1.66	Highly	high excretor
39.	1.02	Highly	high excretor
40.	2.17	Highly	high excretor
41.	0.8	Highly	high excretor
42.	0.9	Highly	high excretor
43.	3.25	Highly	high excretor

44.	2.87	Highly	high excretor
45.	3.98	Massive	high excretor
46.	1.06	Highly	high excretor
47.	4.26	Massive	high excretor
48.	1.42	Highly	high excretor
49.	1.91	Highly	high excretor
50.	1.44	Highly	high excretor
51.	1.54	Highly	high excretor
52.	1.57	Highly	high excretor
53.	0.39	Small	low excretor
54.	1.34	Highly	high excretor
55.	1.44	Highly	high excretor
56.	3.07	Massive	high excretor
57.	0.76	Highly	high excretor
58.	0.77	Highly	high excretor
59.	0.37	Small	low excretor
60.	0.83	Highly	high excretor
61.	0.9	Highly	high excretor
62.	0.59	Highly	high excretor
63.	3.42	Massive	high excretor
64.	0.4	Highly	high excretor
65.	1.35	Highly	high excretor
66.	0.94	Highly	high excretor
67.	1.21	Highly	high excretor
68.	4.32	Massive	high excretor
69.	2.12	Highly	high excretor
70.	1.22	Highly	high excretor
71.	0.88	Highly	high excretor
72.	0.41	Small	low excretor
73.	0.54	Highly	high excretor
74.	0.79	Highly	high excretor
75.	1.04	Highly	high excretor
76.	3.75	Massive	high excretor
77.	1.26	Highly	high excretor
78.	2.12	Highly	high excretor
79.	0.94	Highly	high excretor
80.	0.35	Small	low excretor
81.	1.53	Highly	high excretor
82.	0.57	Highly	high excretor
83.	0.37	Small	low excretor
84.	0.75	Highly	high excretor
85.	1.09	Highly	high excretor
86.	0.64	Highly	high excretor
87.	0.76	Highly	high excretor
88.	4.23	Massive	high excretor
89.	1.21	Highly	high excretor
